# The value of community engagement in the ESTRO–EORTC OligoCare project: A quantification of the in-kind contributions

**DOI:** 10.1016/j.ctro.2026.101296

**Published:** 2026-09-25

**Authors:** Davide Cusumano, Alessandro Scaggion, Yolande Lievens, Enrico Clementel, Sarah Nuyens, Filippo Alongi, Sergi Benavente, Pètra Braam, Piet Dirix, Hossein Hemmatazad, Paul Jeene, Barbara Alicja Jereczek-Fossa, Daniela Greto, Sara Ramella, Jasna But-Hadzic, Umberto Ricardi, Juan Salinas, Marta Scorsetti, Igor Sirak, Karin Stellamans, Joachim Widder, Piet Ost, Matthias Guckenberger

**Affiliations:** a Medical Physics Unit, Mater Olbia Hospital, Olbia (SS), Italy; b Department of Medical Physics, Veneto Institute of Oncology, IOV-IRCCS, Padova, Italy; c Department of Radiation Oncology, Ghent University Hospital; Department of Structure and Repair, Ghent University, Italy; d EORTC, Brussels, Belgium; e Radiation Oncology Division, Sacro Cuore Don Calabria Hospital, 37024 Negrar-Verona, Italy; f University of Brescia, Italy; g Department of Radiation Oncology, Hospital Universitari Vall d'Hebron, Vall d'Hebron Institute of Oncology VHIO, Barcelona, Spain; h Radboud University Medical Center, Nijmegen, the Netherlands; i Iridium Network, Ziekenhuis aan de Stroom (ZAS), Antwerp, Belgium; j Department of Radiation Oncology, Inselspital, Bern University Hospital and University of Bern, Switzerland; k Radiotherapiegroep, Deventer, the Netherlands; l Department of Oncology and Hemato-oncology, University of Milan, Milan, Italy; m Department of Radiation Oncology, IEO European Institute of Oncology IRCCS, Milan, Italy; n Azienda Ospedaliero Universitaria Careggi, Florence, Italy; o Università Campus Bio-Medico di Roma; Fondazione Policlinico Universitario Campus Bio-Medico, Italy; p Division of Radiotherapy, Institute of Oncology Ljubljana, Ljubljana, Slovenia & Faculty of Medicine, University of Ljubljana, Ljubljana, Slovenia; q Department of Oncology, University of Turin, Turin, Italy; r Oncología Radioterapica, Complejo Hospitalario de Cartagena, Murcia, Spain; s Department of Biomedical Sciences, Humanitas University, Pieve Emanuele, Milan, Italy; t Department of Radiotherapy and Radiosurgery, IRCCS Humanitas Research Hospital, Rozzano, Milan, Italy; u University Hospital Hradec Králové & Faculty of Medicine in Hradec, Králové Charles University, Czech Republic; v AZ Groeninge Hospital, Kortrijk, Belgium; w Department of Radiation Oncology, Medical University of Vienna, CCC, Vienna, Vienna, Austria; x Department of Radiation Oncology, University Hospital Zurich, University of Zurich, Zurich, Switzerland

**Keywords:** Cost analysis, In-kind contribution, Real-world data collection

## Abstract

**Background:**

The OligoCare cohort constitutes the largest prospective dataset evaluating metastasis-directed radiotherapy in oligometastatic disease worldwide. Although the project received financial support for central coordination, no financial compensation for the operational involvement of the participating centres was available. This study quantified the in-kind professional contribution provided by the European radiation oncology community.

**Methods:**

In-kind professional contribution was defined as the sum of two components: time invested by clinical staff at enrolling centres for patient management, data collection, and follow-up; and time dedicated by Principal Investigators and Steering Committee members to protocol development, oversight, and coordination. A structured questionnaire was completed by the highest-accruing centres to estimate the time spent by each professional role. Median time requirements were combined with country-specific labour costs derived from OECD and EUROSTAT data to calculate the cost per patient at national level. A 20% overhead was applied to account for indirect activities.

**Results:**

As of August 2025, 3167 patients were enrolled. Questionnaire data from eleven centres (54% of total accrual) showed a median OligoCare-related workload of 218 min per patient (∼3.5 h). The cost per patient varied widely across Europe (79–322€), reflecting national differences in labour costs, resulting in a total in-kind contribution of 626,289€ from enrolling centres. Committee activities accounted for an additional 220,164€. Including overheads, the total in-kind contribution amounted to 1,015,744€.

**Conclusion:**

These findings demonstrate substantial in-kind contributions of the European Radiotherapy community, which were fundamental to the success of OligoCare. Quantifying these efforts supports fair recognition and the development of sustainable funding models for international research.

## Highlights

• European centres carry a major uncompensated workload in OligoCare project.
• This study quantifies the full value of community in-kind contributions.
• Centres invested a mean of 3.5 h per patient in study tasks.
• Per patient cost averaged 300€ based on professional wages.
• Marked cross-country variability emerged due to labour-cost differences.
• Total in-kind contribution was quantified at 1.015.744€ across Europe.

## 1 Introduction

Radiation therapy (RT) is one of the pillars of multidisciplinary cancer care. Nevertheless, collective knowledge is still fragmented regarding how different RT approaches influence clinical endpoints, treatment-related adverse events, and patient-reported quality of life [1], [2], [3], [4], [5].

Randomized clinical trials continue to serve as the gold standard for evidence generation, but their scope is often insufficient to capture the full complexity of clinical practice, the pace at which they produce evidence can be slow and their methodology less appropriate to capture the often incremental and operator-dependent nature of radiotherapy innovations [6], [7]. To address these limitations, complementary strategies that integrate real-world data have emerged in recent years. Prospective observational cohorts, despite inherent constraints, provide unique value by documenting patterns-of-care, assessing effectiveness, and generating hypotheses that can guide the design of future randomized trials of innovative radiotherapy interventions [8].

Within this context, the European Society for Radiotherapy and Oncology (ESTRO) and the European Organisation for Research and Treatment of Cancer (EORTC) jointly launched the E^2^-RADIatE initiative in 2019 (EORTC-ESTRO RADiotherapy Infrastructure for Europe; EORTC 1811 – NCT03818503).

Conceived as a multi-cohort, non-interventional observational platform, E^2^-RADIatE systematically collects real-world data from patients undergoing RT across a broad spectrum of European institutions. The inclusion of both academic and non-academic centres ensures wide participation and data heterogeneity, thereby complementing evidence derived from randomized trials and enabling the exploration of innovative methodological approaches in radiation oncology research [9].

The OligoCare cohort represents the first and largest component of the E^2^-RADIatE program. Its primary objective is to evaluate the role of stereotactic body radiotherapy (SBRT) in patients with oligometastatic disease originating from breast, non-small cell lung, prostate, and colorectal cancers [9].

Since its activation in June 2019, OligoCare has enrolled more than 3000 patients across 67 European centres, establishing the largest prospective dataset worldwide dedicated to metastasis-directed RT in oligometastatic cancer.

In parallel, ESTRO and EORTC introduced in 2020 a shared characterization and classification system for oligometastatic disease, developed through a Delphi consensus involving 20 international experts. This effort aimed to provide the clinical community with a common conceptual framework for a disease state that lies between localized and widely disseminated cancer, as well as a unified nomenclature underpinning the OligoCare project [2], [10]. Interim analyses from OligoCare have already yielded relevant insights into SBRT dose-fractionation patterns, early toxicity profiles, and patient-reported quality-of-life outcomes, while mature overall survival data are anticipated in 2026 [9], [11], [12].

Importantly, although the OligoCare initiative has received financial support from several non-profit sources, this funding was only sufficient to establish and sustain central coordination and platform management of the EORTC. The amount of funding was insufficient to provide direct financial compensation to participating centres for their involvement, although their scientific, clinical, and operational contributions were fundamental to the success of the project.

The present analysis aims to quantify these in-kind contributions, providing a transparent assessment of the collective effort invested by the European radiation oncology community. Such quantification is essential to inform future funding models and to ensure that academic and clinical engagement is adequately recognized in large-scale collaborative research initiatives. In addition, a secondary more generalised analysis was performed to estimate the additional costs that should be considered when designing a European multicentre project intended to equitably compensate the involvement of participating accrual centres.

## 2 Methods

To estimate the in-kind contribution associated with the OligoCare project, the number of patients enrolled in the OligoCare database up to August 2025 was extracted, together with information on the corresponding enrolling centres. The highest-accruing centres within the consortium were subsequently contacted and invited to participate in the present analysis, aiming to include at least one centre per participating European country.

For each country, the data provided by the designated representative centre were deemed indicative of national practice; accordingly, each patient originating from that country was assessed on the basis of those representative data.

Uncompensated professional effort was defined as the sum of two main components: (i) the time invested by clinical staff at each accrual centre for patient management, data collection, and follow-up activities (Site-Level Contribution); and (ii) the time dedicated by Principal Investigators (PIs) and Steering Committee members to protocol development, scientific oversight, and study coordination (Central Coordination Contribution).

To account for indirect and non-itemised activities, such as institutional support (e.g., office space and utilities), coordination tasks (e.g., email communication and administrative work), training for the use of centralised data-collection platforms, and travel time, a 20% overhead was applied to the final cost estimate [13]. In summary, the total in-kind contribution (IKC) was calculated as:

$$\mathrm{IKC}=1.2\times \left(\mathrm{SLC}+\mathrm{CCC}\right)$$

where SLC denotes Site-Level Contribution and CCC denotes Central Coordination Contribution.

## 3 Site-level contribution collection

Site-level contributions were estimated by quantifying the per-patient time required from staff at each participating centre for study-related activities, including clinical data collection, data entry into the OligoCare platform, and follow-up visits. A structured questionnaire was administered to collect detailed information on the additional time required, stratified by professional role and activity type.

Each centre reported the per-patient time contributed by three professional categories (Radiation Oncologists, Medical Physicists, and study Nurses or Data Managers) for the following activities: initial patient information, treatment and planning data collection and follow-up visits.

To quantify the time required to manage an OligoCare patient (τ_pat_), the initial patient information and treatment-plan data collection were consolidated into the single baseline data collection (BDC) value, representing all activities performed once per patient. Follow-up (FUP) captured the additional time required at each subsequent follow-up visit. The total time per single patient was then calculated as:

$$\tau_{\mathrm{pat}}=t_{\mathrm{BDC}}+t_{\mathrm{FUP}}\times n_{\mathrm{FUP}}$$

where nFUP represents the average number of follow-up visits per patient, derived from EORTC registry data. Based on the OligoCare registry at the 08/2025 data cut-off, $n_{\mathrm{FUP}}\;$= 3 was applied.

The median of the values reported by the participating centres was used as the reference time for each professional role. Medians were computed over centres reporting non-zero involvement, and a role-activity combination was retained only where at least three centres reported non-zero time. The economic value of professional time was estimated as the full hourly cost to the employer. For each country, the average gross annual remuneration of salaried specialist medical practitioners and of hospital nurses was obtained from the Organisation for Economic Co-operation and Development (OECD) Health Statistics remuneration dataset (worker status: employees; national currency, current prices), at the most recent vintage available for that country at the time of extraction [14]. Figures reported in national currency were converted to euro at the European Central Bank annual average reference rate for the corresponding year.

Because the OECD definition excludes social contributions payable by the employer, gross remuneration *S* was converted to the total annual cost to the employer as *C = S / (1 - s)*, where *s* is the country-specific share of non-wage costs in total labour costs published by EUROSTAT (dataset *lc_lci_lev*, whole economy; accessed on 22 August 2025) [15]. For the United Kingdom and Switzerland, for which this share is not published, the EU-27 average of 24.7% was applied.

Annual costs were converted into hourly rates assuming 1760 effective working hours per year (40 h per week over 44 effective working weeks, net of statutory leave, public holidays and training), applied uniformly to all countries and professional roles so as not to affect comparisons between countries. Nurses and Data Managers were assigned the same hourly cost, no harmonised European wage statistics being published for clinical research data managers, while Medical Physicists were assumed to correspond to 80% of Radiation Oncologists. The OECD dataset reports no salaried specialist remuneration for Austria and, for Switzerland, self-employed specialists only, while the United Kingdom (UK) series (NHS Digital Electronic Staff Record, England only, classified as experimental) ends in 2020.

The complete parameter set, including the reference year and source for every country and professional role, is reported in Supplementary Table S1. The effect of the heterogeneous salary reference years was assessed by repeating the entire calculation with all labour costs re-anchored on 2023 (Supplementary Table S3). Uncertainty in the pooled time estimates was quantified using a robust dispersion metric derived from the interquartile range.

## 4 Central coordination contribution estimation

Central Coordination Contribution was derived from EORTC records documenting the number of Steering Committee meetings held over the years, as well as the time invested by the PI and co-PI in protocol development, monitoring, coordination, and wrap-up activities. The estimation distinguished between the cost attributable to PI and co-PI time and the cost associated with Steering Committee members. As all committee members hold senior positions within their departments, their hourly labour cost was assumed to be twice the estimated hourly cost of the corresponding professional role in their respective countries, based on OECD data.

## 5 Results

At the time of the data collection (August 2025) 3.167 patients were enrolled in OligoCare. This analysis included data obtained from 11 centres: three from Belgium, two from Italy, and one from The Netherlands, Switzerland, Slovenia, Czech Republic, Austria, and Spain, representing a total of 1709 patients (54%).

Fig. 1 illustrates both the total number of patients enrolled in the OligoCare project and the subset included in the present questionnaire-based analysis. Fig. 2 summarises the times reported by each participating centre for the two main activities and for all professional categories.

**Fig. 1:**
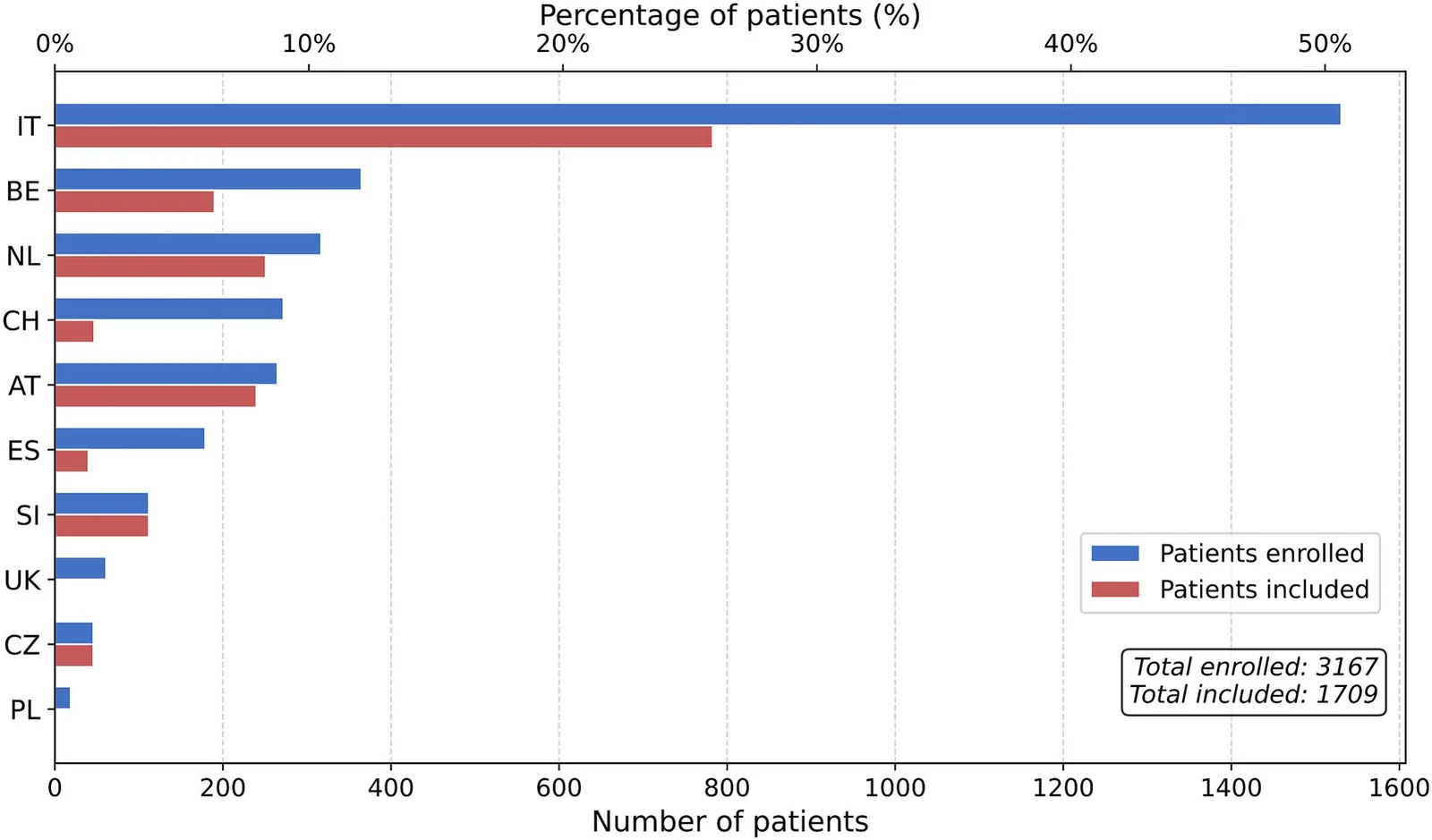
- Geographic distribution of patients enrolled and included in the Oligocare study across participating countries. Blue and red bars represent the percentage number of patients enrolled in the cohort and included in this evaluation, respectively. Italy (IT) contributed the highest number of patients, followed by Belgium (BE) and the Netherlands (NL). Other participating countries include Switzerland (CH), Austria (AT), Spain (ES), Slovenia (SI), Great Britain (UK), Czech Republic (CZ), and Poland (PL). (For interpretation of the references to colour in this figure legend, the reader is referred to the web version of this article.)

**Fig. 2:**
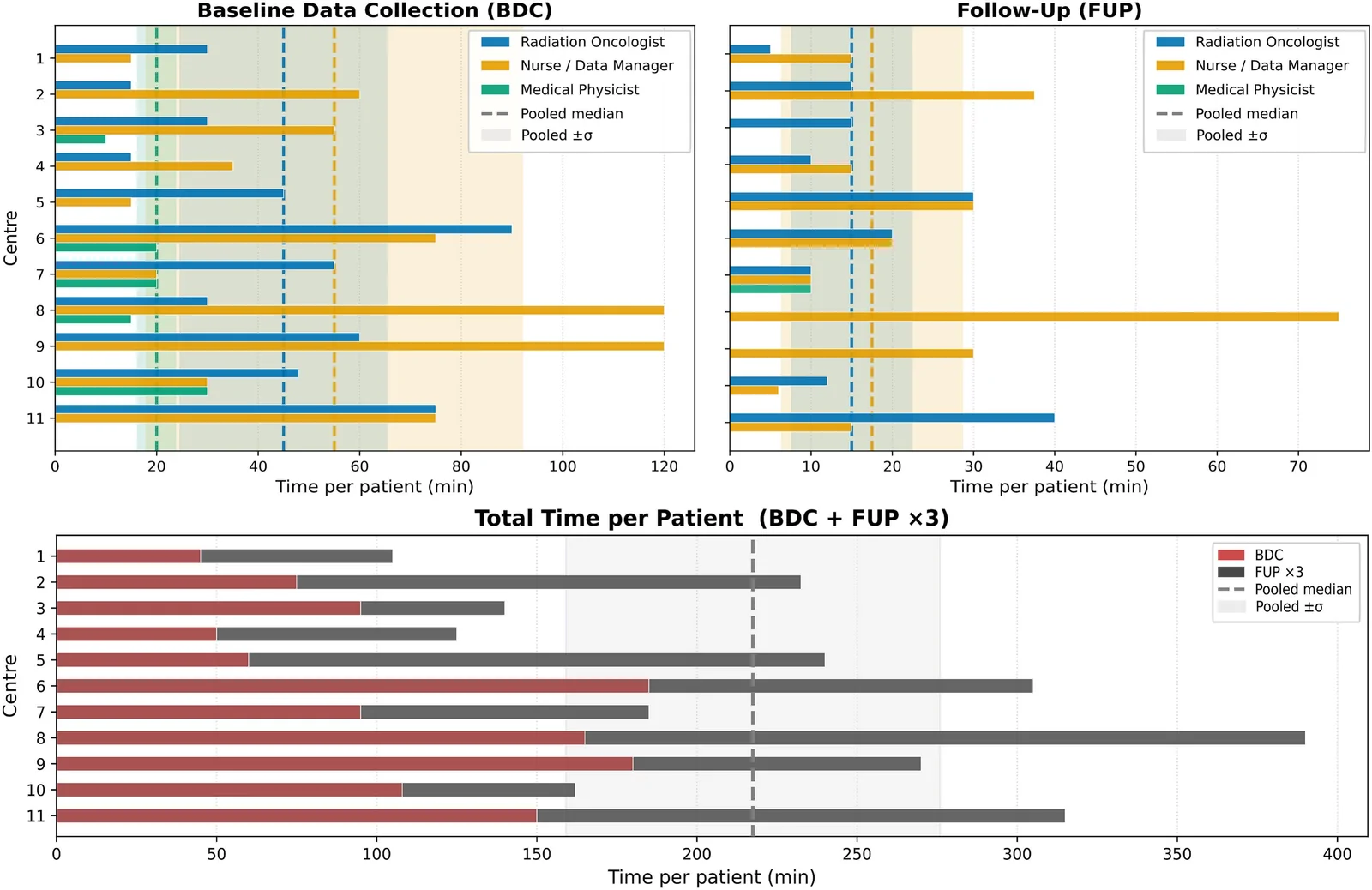
— Distribution of time dedicated to the two key activities per each single patient by three professional roles (Data Manager/Nurse, Medical Physicist, Radiation Oncologist) across 11 European centres. Times are expressed in minutes (min); follow-up times refer to a single visit. Pooled median values for each professional role are reported with dashed vertical lines, while shadowed areas represent the standard deviation.

Pooled median values are reported in Table 1: BDC was the most time-consuming activity, with pooled medians of 45 min for Radiation Oncologists, 55 min for Nurses/Data Managers, and 20 min for Medical Physicists. FUP visits required approximately one-third of the BDC time (15 min for Radiation Oncologists and 17.5 min for Nurses/Data Managers). The workload distribution between Radiation Oncologists and Nurses/Data Managers was relatively balanced, with Nurses/Data Managers contributing slightly more time across both activities. Medical Physicists had a more limited involvement, contributing only to BDC: follow-up time for Medical Physicists was reported by a single centre and was therefore excluded.

**Table 1:** – Distribution of the time expressed in minutes (median, minimum, maximum and interquartile range) required for baseline data collection (t_BDC_), follow-up (t_FUP_), and total time (τ). Values are reported separately for each professional role and as cumulative totals. Medians, minima, maxima and IQR are computed over all eleven centres, including those reporting no involvement of a given role. The values used in the cost model are the medians over centres reporting non-zero time, retained only where at least three centres did so.

Time (min)	Value	Professional role			
		Radiation Oncologist	Nurse/ Data Manager	Medical Physicist	Cumulative Time
t_BDC_	Median	45	55	0	95
	Value used in model	45	55	20	120
	Min	15	15	0	45
	Max	90	120	30	185
	IQR	27,5	50	17.5	90
t_FUP_	Median	12	15	0	30
	Value used in model	15	17.5	n.a.	32.5
	Min	0	0	0	15
	Max	40	75	10	75
	IQR	10	17.5	0	31.2
τ	Median	75	105	0	232.5
	Value used in model	90	107.5	20	217.5
	Min	30	48	0	105
	Max	195	345	50	390
	IQR	57.5	96.3	17.5	136.5

The total pooled time per enrolled patient was estimated at 217.5 min (range of centre-level totals: 105–390 min), corresponding to roughly 3.5 h. Substantial inter-centre variability emerged across all activity–profession combinations, as reflected by the wide dispersion of centre-specific values around the pooled estimates. For instance, BDC times ranged from 15 to 90 min for Radiation Oncologists, from 0 to 30 min for Medical Physicists, and from 15 to 120 min for Nurses/Data Managers, depending on the enrolling centre. Notably, in five centres Medical Physicists were not involved in the process at all. A similarly broad distribution was observed for follow-up visits, which ranged from 5 to 40 min for Radiation Oncologists and from 10 to 75 min for Nurses. Only one centre in Spain reported additional Medical Physicist time during OligoCare follow-up (10 min). Interestingly, in 2 out of 11 centres (one Dutch and one Swiss centre), clinicians did not allocate any additional time for OligoCare follow-up visits; in these settings, the extra time required for follow-up was borne exclusively by Nurses.

Table 2 presents the estimated yearly and hourly labour costs for Radiation Oncologists and Nurses/Data Managers, inclusive of non-wage cost components. Across Europe, the median hourly cost was approximately 72€ for Radiation Oncologists and 31€ for Nurses. Comparing these data to the self-reported ones by the participating centres we estimated a variability of 55.4% for Radiation Oncologist and 66% for Nurse/Data Manager yearly costs.

**Table 2:** - Estimated yearly and hourly labour costs for Radiation Oncologists and Nurses/Data Manager for the countries which enrolled patient in OligoCare based on OECD Health Statistics and EUROSTAT data. Annual cost = hourly cost x 1760 h/year. Hourly Medical Physicist cost = 0.80 x hourly Radiation Oncologist cost. The reference year and source for each country are given in Supplementary Table S1 [14], [15].

Country	Yearly labour cost		Hourly labour cost	
	Radiation Oncologist	Nurse/Data Manager	Radiation Oncologist	Nurse/Data Manager
Italy	110.000 €	42.384 €	62 €	24 €
Belgium	170.000 €	90.000 €	97 €	51 €
The Netherlands	207.778 €	77.778 €	118 €	44 €
Switzerland	219.944 €	99.867 €	125 €	57 €
Austria	142.222 €	61.111 €	81 €	35 €
Spain	110.000 €	52.222 €	62 €	30 €
Slovenia	71.111 €	37.778 €	40 €	21 €
United Kingdom	181.355 €	57.222 €	103 €	33 €
Czech Republic	65.600 €	30.841 €	37 €	18 €
Poland	50.400 €	28.267 €	29 €	16 €

Combining the pooled median time with the nation-specific estimated hourly labour costs allows computation of the final per-patient contribution (PPC) for each country, as reported in Table 3.

**Table 3:** – Summary of the per-patient contribution (PPC) for each accruing country, including total patient accrual and its proportion of the overall cohort, the estimated PPC, the corresponding total in-kind contribution, and each country's share of the overall in-kind contribution. Countries are listed in descending order of their percentage of accrued patients. The total in-kind contribution across the ten countries is 626.289€.

Country	Number of patients enrolled	Percentage of patients enrolled	Per patient cost (PPC)	In-Kind Contribution	Percentage of In-Kind
Italy	1531	48.34%	154 €	235.106 €	37.54%
Belgium	365	11.53%	262 €	95.726 €	15.28%
The Netherlands	317	10.01%	288 €	91.214 €	14.56%
Switzerland	272	8.59%	322 €	87.704 €	14.00%
Austria	265	8.37%	205 €	54.317 €	8.67%
Spain	179	5.65%	164 €	29.281 €	4.68%
Slovenia	112	3.54%	110 €	12.302 €	1.96%
United Kingdom	61	1.93%	240 €	14.658 €	2.34%
Czech Republic	46	1.45%	97 €	4.473 €	0.71%
Poland	19	0.60%	79 €	1.508 €	0.24%

PPC ranged from 79€ (Poland) to 322€ (Switzerland), a four-fold difference driven primarily by national labour cost disparities. The total contribution from the enrolling centres attributable to protocol-related per-patient activities amounted to 626,289€.

As for the committee cost estimation, the Steering Committee consisted of two cohort coordinators (both Radiation Oncologists) and ten additional members (three Medical Physicists and seven Radiation Oncologists). Three EORTC staff members also participated but were not included in the contribution evaluation, as their contribution was already covered by institutional funding. The full composition, including country and hourly labour cost (estimated as twice the national labour cost), is reported in Table 4.

**Table 4:** - Composition of the Steering Committee, including professional roles (Radiation Oncologist or Medical Physicist and average hourly wage estimates.

Role	Radiation Oncologists	Medical Physicists	Hourly Wage	In-Kind Contribution
Cohort Coordinator	2	0	222 €	127.584 €
ESTRO representative	1	1	159 €	19.080 €
TwiCs Expert	2	2	145 €	34.740 €
Top Recruiter	4	0	162 €	38.760 €

For the estimation of the total cost, six years of activity were considered for the coordinators, whereas five years were included for the remaining Steering Committee members. According to the EORTC registry, the estimated engagement corresponded to 1 h per month for each Steering Committee member and 4 h per month for each coordinator. This resulted in a total of 288 h per coordinator (576 h in total) and 60 h per Steering Committee member (600 h in total). Based on the corresponding hourly wages, the total Central Coordination Contribution was estimated at 220,164€.

Summing the total contribution from the enrolling centres (626,289€) with the Central Coordination Contribution and applying a 20% overhead results in a grand total of 1,015,744€.

## 6 Discussion

Although radiotherapy remains a mainstay of cancer treatment, it faces a landscape of diminishing funding for clinical research that is particularly acute within the radiation oncology sector. Despite these financial constraints, the EORTC ESTRO OligoCare project accomplished the world's largest prospective registry in the field of oligometastatic disease, thanks to the community's strong commitment and collaborative spirit. Cost quantification of clinical research is considered to contribute to sustainability of such efforts by a fair recognition of the significant effort and resources invested by participating centers [16], [17].

The present analysis provides a comprehensive evaluation of the in-kind professional contribution offered by the European radiation oncology community to the OligoCare cohort, showing that the enrolment-weighted mean cost is €198 per patient and that the cumulative value of the professionals' in-kind contribution approaches one million euros, underscoring the substantial collective investment underpinning the OligoCare project.

Although these values are considerable, they remain markedly lower than those typically associated with conventional clinical trials that do not rely on real-world data. For example, in oncology drug-development interventional trials, per-patient costs are orders of magnitude higher: a recent JAMA analysis estimated per-patient costs ranging from 23,515$ to 103,344$, depending on the study phase [18]. This comparison highlights the cost-effectiveness of large-scale real-world data initiatives such as OligoCare in the field of radiation oncology.

A key finding of this study is the pronounced inter-institutional variability in the additional time devoted to the inclusion of each patient in the Oligocare program, ranging from 105 to 390 min across European centres. This heterogeneity likely reflects differences in workflow organisation, data-collection procedures, staffing models, and institutional infrastructures, as well as the intrinsic diversity of European healthcare systems. This variability highlights an important opportunity: centres with higher time investments may benefit from the practices of more time-efficient institutions, supporting process optimization, training, harmonization, and ultimately a more sustainable implementation of large-scale real-world data initiatives.

With a median time investment of 3.5 h per patient (217.5 min), the cumulative workload for high-accruing centres is substantial, corresponding to an estimated 626,289€ for the entire cohort of 3167 patients. Centres providing the largest in-kind contributions also derived important benefits from their engagement. Participation enabled meaningful institutional benchmarking, facilitated community building within OligoCare, and supported the development of multiple side projects and interim analyses. The structured coordination provided by EORTC further enhanced the scientific value and long-term cohesion of the network. Another important result of this study is the high per-patient cost variability which emerged, ranging from around 100€ or less in Eastern European countries (Slovenia, Czech Republic, and Poland) to around 300€ or more in Switzerland and The Netherlands: this pattern mirrors the well-documented differences in labour costs across Europe and has direct implications for the design of future multicentre studies [14], [19].

Equitable compensation models must account for national wage structures, which inherently increase the cost of participation for centres in higher-income countries. In this context, three distinct aggregate figures emerge and serve different planning purposes. The unweighted mean of the ten national per-patient costs is 192€, the figure relevant to a study seeking to compensate participating centres equitably across countries. The enrolment-weighted mean is 198€, which reflects the actual cost structure of OligoCare and is pulled below the unweighted mean by the 48.3% share of accrual contributed by Italy, a comparatively low-cost country. Including central coordination and overheads, the total in-kind contribution corresponds to 321€ per enrolled patient, the appropriate figure when budgeting a prospective multicentre study end to end.

These figures must not be conflated: applying the third to an Italy-weighted cohort would overestimate site-level costs by roughly two thirds. For study design we would recommend the country-specific values of Table 3 in preference to any single European average. Alternatively, as suggested by the country-specific values reported in Table 3, tailored reimbursement schemes could be developed to reflect national economic contexts more accurately.

The contribution of Coordinators and Steering Committee members also deserves recognition: their uncompensated effort over six years is estimated at 220,164€. Although this estimate relies on assumptions (e.g., seniority-adjusted wages), it provides a conservative approximation of the economic value associated with scientific oversight, protocol development, and governance, activities essential for ensuring methodological rigour, data quality, and long-term project continuity, yet rarely budgeted in multicentre studies. When overheads are included, the total in-kind contribution of the community reaches 1,015,744€, corresponding to 321€ per enrolled patient.

Several limitations must be acknowledged. Time-activity data were collected only from the highest-accruing centres, and the assumption of homogeneous time requirements within each country may overlook regional variability. Some cost assumptions, such as equating Nurse and data-manager wages, estimating Medical Physicist salaries as 80% of Radiation Oncologists, or applying a uniform annual working time of 1760 h, may not hold across all institutions. Wages may also vary substantially within the same centre, depending on seniority, expertise, and contractual arrangements, as well as within one country, depending on regional reimbursement models or the type of institution.

Labour cost coverage was also incomplete for three countries. In the OECD dataset, Austria reports no salaried specialist remuneration, while Switzerland provides data only for self-employed specialists. The UK series refers to England alone, is classified as experimental, ends in 2020, and does not account for the post-Brexit context. No UK centre responded to the questionnaire; therefore, the UK estimate in this study is entirely model-derived. Its overall impact is limited, however, as the UK contributes only 1.9% of total accrual, and even a 30% variation in the assumed UK hourly costs would change the overall estimate by less than 0.6%.

Nonetheless, these limitations are unlikely to alter the overall conclusion: the community's in-kind contribution represents a major economic component of the OligoCare project and is a critical enabler of its scientific success.

A further question we were unable to address is whether the time invested per patient declined within a centre over the recruitment period. Such a learning-curve effect is plausible, as centres consolidate workflows, train staff and adapt local procedures to the platform, and it would have direct implications for how the cost of a prospective registry should be budgeted over its lifetime. Our questionnaire, however, asked for a single current estimate per role and activity, and no time-stamped record of study-related staff effort was collected, so the present data cannot resolve it. We would recommend that future real-world data programmes embed lightweight periodic effort reporting, for example a brief questionnaire repeated annually, so that this effect can be quantified prospectively rather than reconstructed after the fact.

## 7 Conclusion

The present analysis highlights the strategic importance of systematically recognising and valuing in-kind contributions within academic clinical research. By integrating detailed time-investment data from high-accruing centres with country-specific labour cost estimates, this study demonstrates that community engagement represents a substantial and indispensable component of the resources required to sustain a multi-institutional research initiative. As real-world evidence programmes continue to expand and the demand for high-quality prospective data grows, transparent accounting of these contributions will be essential to ensure fair resource allocation, reinforce collaborative networks, and support the long-term sustainability of European research infrastructures such as E^2^-RADIatE.

## CRediT authorship contribution statement

**Davide Cusumano:** Writing – original draft, Formal analysis. **Alessandro Scaggion:** Writing – original draft, Data curation. **Yolande Lievens:** Conceptualization. **Enrico Clementel:** Resources. **Sarah Nuyens:** Project administration. **Filippo Alongi:** Resources. **Sergi Benavente:** Resources. **Pètra Braam:** Resources. **Piet Dirix:** Resources. **Hossein Hemmatazad:** Resources. **Paul Jeene:** Resources. **Barbara Alicja Jereczek-Fossa:** Supervision. **Daniela Greto:** Resources. **Sara Ramella:** Resources. **Jasna But-Hadzic:** Resources. **Umberto Ricardi:** Resources. **Juan Salinas:** Resources. **Marta Scorsetti:** Resources. **Igor Sirak:** Resources. **Karin Stellamans:** Resources. **Joachim Widder:** Resources. **Piet Ost:** Conceptualization. **Matthias Guckenberger:** Writing – review & editing, Supervision, Conceptualization.

## Declaration of competing interest

The authors declare the following financial interests/personal relationships which may be considered as potential competing interests: [Prof. Barbara Jereczek‑Fossa reports receiving personal fees/honoraria from Janssen‑Cilag, Bayer, Ipsen, Accuray, Astellas Pharma, AstraZeneca, Pfizer, Accord, Novartis, Seagen, Daiichi Sankyo, Radformation, Tecnologie Avanzate, IBA, and Recordati, all outside the submitted work. She also reports institutional grants and research funding from ACCURAY, IBA, AIRC (Italian Association for Cancer Research), and Fondazione IEO‑CCM (Istituto Europeo di Oncologia – Centro Cardiologico Monzino), all outside the submitted work.

Prof. Umberto Ricardi reports receiving honoraria from AstraZeneca and Servier.

Prof. Filippo Alongi reports receiving speaker honoraria, consultancy fees, and research grants from Varian, Elekta, and Brainlab.

Prof. Yolande Lievens reports receiving grant funding from Varian Medical Systems Inc.

Prof. Matthias Guckenberger reports receiving research funding from Siemens Healthineers and consulting fees as an advisory board member for Johnson & Johnson, Siemens Healthineers, and AstraZeneca. He also serves as President of ESTRO.

Prof. Piet Ost has served as a consultant or advisor for Bayer, Janssen, Novartis, AstraZeneca, Telix Pharmaceuticals, and Astellas Pharma. He has received honoraria from Curium Pharma, Recordati, and Micropos. His institution receives research funding from Bayer. He has served on a paid IDMC for Telix Pharmaceuticals (ProstACT GLOBAL trial). He serves as an unpaid IDMC member for the PEARLS, PEACE‑6 OligoPresto, STAMPEDE2, and STAR‑TRAP trials. He holds leadership positions within ESTRO (Board Member, Chair of the Urology Focus Group) and EORTC (Chair of the Radiation Oncology Scientific Council). He previously served on an unpaid advisory board for ICR UK.

Prof. Joachim Widder reports that his institution receives research grants from Brainlab, Elekta, Philips, RaySearch, and Therapanacea.

Prof. Jasna But Hadžić received speaker honoraria from AstraZeneca, Roche, Medison Pharma and Ewopharma. All the other authors declare no known competing financial interests or personal relationships that could have appeared to influence the work reported in this paper].
