# Supplementary material for: The value of community engagement in the ESTRO–EORTC OligoCare project: A quantification of the in-kind contributions
Source: Clin Transl Radiat Oncol. 2026 Sep 25;62:101296. doi: 10.1016/j.ctro.2026.101296 (PMC13635552; doi:10.1016/j.ctro.2026.101296)
Supplement: Supplementary file 1 [file mmc1.docx]

The value of community engagement in the ESTRO–EORTC OligoCare project: a quantification of the in-kind contributions

**Supplementary Material**

**S1 - Derivation of country-specific hourly labour costs**

For each country and professional role, the hourly cost to the employer was obtained as

***c = [S / (1 − s)] / H,***

where ***S*** is the average gross annual remuneration, ***s*** the share of non-wage costs in total labour costs, and ***H*** = 1,760 the effective working hours per year (40 h/week × 44 effective working weeks). Gross remuneration was taken from the OECD Health Statistics remuneration dataset (worker status: employees; national currency, current prices), converted to euro at the ECB annual average reference rate for the corresponding year. Non-wage cost shares are from ***EUROSTAT lc_lci_lev*** data series (whole economy, 2024). Hourly Medical Physicist cost was set at 0.80 × hourly Radiation Oncologist cost.

***TABLE S1 – Details of the derivation of country-specific hourly labour costs***

| **Country** | **Role** | **Gross annual S (€)** | **Reference year *** | **Non-wage share s** | **Employer cost C = S/(1−s) (€)** | **Hourly cost c = C/H (€/h)** | **Source of S** |
| --- | --- | --- | --- | --- | --- | --- | --- |
| Italy | RO | 79,310 | 2023 | 27.9% | 110,000 | 62.50 | OECD Health Statistics |
| Italy | N/DM | 30,559 | 2023 | 27.9% | 42,384 | 24.08 | OECD Health Statistics |
| Belgium | RO | 130,900 | 2022 | 23.0% | 170,000 | 96.59 | OECD Health Statistics |
| Belgium | N/DM | 69,300 | 2018 | 23.0% | 90,000 | 51.14 | OECD Health Statistics |
| The Netherlands | RO | 156,872 | 2019 | 24.5% | 207,778 | 118.06 | OECD Health Statistics |
| The Netherlands | N/DM | 58,722 | 2020 | 24.5% | 77,778 | 44.19 | OECD Health Statistics |
| *Switzerland* | *RO* | *165,618* | *n.a.* | *24.7%*** | *219,944* | *124.97* | *national / secondary **** |
| *Switzerland* | *N/DM* | *75,200* | *2022* | *24.7% *** | *99,867* | *56.74* | *OECD Health Statistics* |
| *Austria* | *RO* | *103,538* | *n.a.* | *27.2%* | *142,222* | *80.81* | *national / secondary **** |
| *Austria* | *N/DM* | *44,489* | *n.a.* | *27.2%* | *61,111* | *34.72* | *national / secondary **** |
| Spain | RO | 81,950 | 2023 | 25.5% | 110,000 | 62.50 | OECD Health Statistics |
| Spain | N/DM | 38,906 | 2019 | 25.5% | 52,222 | 29.67 | OECD Health Statistics |
| Slovenia | RO | 61,084 | 2022 | 14.1% | 71,111 | 40.40 | OECD Health Statistics |
| Slovenia | N/DM | 32,451 | 2020 | 14.1% | 37,778 | 21.46 | OECD Health Statistics |
| *United Kingdom* | *RO* | *136,561* | *2020* | *24.7% *** | *181,355* | *103.04* | *OECD Health Statistics ***** |
| *United Kingdom* | *N/DM* | *43,088* | *2023* | *24.7% *** | *57,222* | *32.51* | *OECD Health Statistics* |
| Czech Republic | RO | 49,528 | 2021 | 24.5% | 65,600 | 37.27 | OECD Health Statistics |
| Czech Republic | N/DM | 23,285 | 2019 | 24.5% | 30,841 | 17.52 | OECD Health Statistics |
| Poland | RO | 41,328 | 2021 ******* | 18.0% | 50,400 | 28.64 | OECD Health Statistics |
| Poland | N/DM | 23,179 | 2021 ******* | 18.0% | 28,267 | 16.06 | OECD Health Statistics |

*RO = Radiation Oncologist (salaried specialist medical practitioner). N/DM = Nurse / Data Manager (hospital nurse). Values are shown rounded; all calculations were performed at full precision.*

** Reference year of the OECD extraction, which differs between countries and professional roles because national reporting timetables and series breaks differ; the most recent vintage available for each country at the time of extraction (22 August 2025) was used. Gross annual remuneration is reported as entered in the analysis; minor differences from the currently published OECD figures may arise from revisions to the series subsequent to the extraction date. Table S3 reports the effect of re-anchoring the whole analysis on a single, more recent reference year.*

*** EUROSTAT does not publish the non-wage cost share for Switzerland or the United Kingdom; the EU-27 average of 24.7% was applied.*

**** The OECD dataset reports no salaried specialist remuneration for Austria and, for Switzerland, self-employed specialists only. Values for these countries were derived from national and secondary sources aligned with the OECD framework.*

***** The United Kingdom salaried specialist series (NHS Digital Electronic Staff Record; England only; classified by the OECD as experimental) ends in 2020, which is therefore the reference year used.*

****** The Polish Structure of Earnings survey is biennial, with waves in 2020 and 2022; the 2021 value is the mean of the two adjacent waves.*

**S2 - Sensitivity to the annual working time assumption**

Every cost in the model has the form (time in minutes) × (hourly cost) / 60, and every hourly cost is obtained as ***c = C / H***. The assumed annual working time ***H*** therefore enters the model only as a single multiplicative constant, applied identically to all countries and professional roles, so the entire estimate scales inversely with ***H***. Data in the manuscript are reported for ***H₀ = 1,760 h/year*** (40 h/week × 44 weeks), a simple sensitivity analysis can thus be conducted computing the new In-Kind Contribution as

***IKC(H) = IKC(H₀) × H₀ / H.***

A relative change in ***H*** produces an equal and opposite relative change in the estimate. Over the interval 1,680–1,840 h/year pre-specified in the analysis plan (from 40 h/week × 42 weeks to 40 h/week × 46 weeks), the Site-Level Contribution ranges from €656,112 to €599,059 and the total in-kind contribution from €1,064,112 to €971,581, that is +4.8% to −4.3% around the base case.

Because ***H*** is a common factor, it cancels in every ratio derived from the model. The share of each country in the total, the relative contribution of each professional role, the ranking of countries by per-patient cost and all comparisons between settings are invariant with respect to ***H***; only the absolute level of the estimate is affected.

**S3 — Sensitivity to the salary reference year**

The primary analysis uses, for each country and professional role, the most recent OECD vintage available at the time of extraction, which ranges from 2018 to 2023 because national reporting timetables and series breaks differ. To test whether this heterogeneity affects the findings, the entire analysis was repeated with all labour costs re-anchored on 2023, the most recent year for which the OECD series is available across the majority of participating countries. Poland was anchored on 2022, the most recent wave of its biennial Structure of Earnings survey. Four values have no 2023 OECD equivalent (Austrian Radiation Oncologists and Nurses/Data Managers, Swiss Radiation Oncologists and United Kingdom Radiation Oncologists) and were uprated to 2023 using country-specific wage indices.

***TABLE S3 – Sensitivity to the salary reference year***

| **Country** | **PPC, primary analysis** | **PPC, 2023-anchored** | **Change** | **In-kind, 2023-anchored** |
| --- | --- | --- | --- | --- |
| Italy | 154 € | 154 € | +0.0% | 235,161 € |
| Belgium | 262 € | 297 € | +13.2% | 108,343 € |
| The Netherlands | 288 € | 334 € | +15.9% | 105,733 € |
| Switzerland | 322 € | 348 € | +7.8% | 94,527 € |
| Austria | 205 € | 217 € | +5.7% | 57,405 € |
| Spain | 164 € | 169 € | +3.3% | 30,244 € |
| Slovenia | 110 € | 128 € | +16.4% | 14,321 € |
| United Kingdom | 240 € | 265 € | +10.4% | 16,186 € |
| Czech Republic | 97 € | 117 € | +20.8% | 5,402 € |
| Poland | 79 € | 87 € | +10.2% | 1,662 € |
| **Total in-kind contribution** | **626,289 €** | **668,984 €** | **+6.8%** | **—** |

*Uprating factors for the four values without a 2023 OECD equivalent: Switzerland ×1.065 and United Kingdom ×1.135, from the growth of the respective OECD hospital nurse series (2022–2023 and 2020–2023); Austria ×1.057, from the EUROSTAT hourly labour cost index (2022–2023).*

Re-anchoring raises the Site-Level Contribution by 6.8% and the total in-kind contribution by 5.0%, from €1,015,744 to €1,066,977, corresponding to €337 rather than €321 per enrolled patient. The relative ranking of countries is unchanged and Switzerland remains the highest-cost setting. The direction and magnitude of the effect reflect wage growth across Europe between the two vintages rather than any change in methodology; the primary analysis is therefore conservative.

**S4 — Internal validation of the labour cost estimates**

Nine centres reported their own hourly labour costs in the questionnaire. For each centre ***i*** and role ***p***, the relative deviation of the self-reported value from the model estimate was computed as ***δ = (h_survey − h_model) / h_model.*** Because these deviations are strongly skewed by isolated extreme values, central tendency and dispersion were summarised using the median and the median absolute deviation (MAD), with the coefficient of variation estimated as ***CV = 1.4826 × MAD***. This is the source of the variability figures reported in the Results.

***S4 — Internal validation of the labour cost estimates***

| **Professional role** | **n** | **Relative deviations δ (%)** | **Median δ** | **MAD** | **CV = 1.4826 × MAD** |
| --- | --- | --- | --- | --- | --- |
| Radiation Oncologist | 9 | −49, −49, −33, −33, +5, +9, +10, +29, +91 | +4.6% | 37.4% | 55.4% |
| Nurse / Data Manager | 9 | −44, −43, −17, −17, +2, +22, +64, +66, +183 | +1.6% | 44.5% | 66.0% |
| Medical Physicist | 10 | −100, −50, −41, −33, −17, −6, +14, +29, +36, +85 | −11.7% | 34.1% | 50.5% |

A median deviation close to zero indicates that the model estimate is unbiased on average with respect to the values centres report for themselves. For Nurses/Data Managers the median deviation of +1.6% supports the assumption that nurse wage statistics are an adequate proxy for data managers; for Medical Physicists the median deviation of −11.7% indicates that the assumption of 80% of the Radiation Oncologist cost is mildly conservative in the direction of over-estimation.

No United Kingdom centre responded to the questionnaire, so the Radiation Oncologist and Medical Physicist estimates are independent of the United Kingdom labour cost values.

Medical Physicist hourly costs are a fixed fraction of Radiation Oncologist costs, so the two sets of deviations are not statistically independent.
